# Supplementary material for: Novel Nickel(II), Palladium(II), and Platinum(II) Complexes with O,S Bidendate Cinnamic Acid Ester Derivatives: An In Vitro Cytotoxic Comparison to Ruthenium(II) and Osmium(II) Analogues
Source: Int J Mol Sci. 2022 Jun 15;23(12):6669. doi: 10.3390/ijms23126669 (PMC9224311; doi:10.3390/ijms23126669)
Supplement: Supplementary file 1 [file ijms-23-06669-s001.zip › ijms-1668983-supplementary.pdf]

Supplementary Part

Novel Nickel(II), Palladium(II), and Platinum(II) complexes with O,S-bidendate cinnamic acid ester derivatives: An *in vitro* cytotoxic comparison to Ruthenium(II) and Osmium(II) Analogues

Jana Hildebrandt[1,2], Norman Häfner[2], Helmar Görls[1], Marie-Christin Barth[1], Matthias Dürst[2], Ingo B. Runnebaum[2], Wolfgang Wiegand[1]

[1] Institut für Anorganische und Analytische Chemie Friedrich-Schiller Universität Jena, Humboldtstraße 8, 07743 Jena, Germany

[2] Department of Gynecology, Jena University Hospital – Friedrich-Schiller University Jena

Additional Characterization

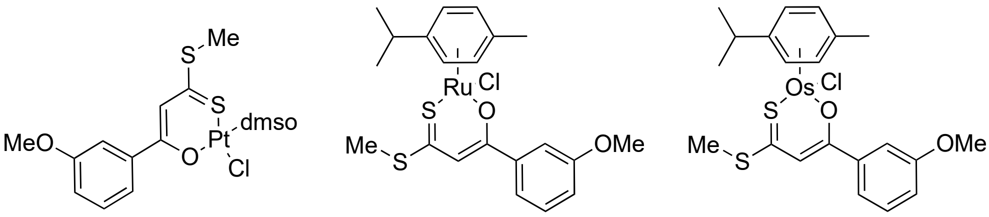

Ptdmso2 Ru2 Os2

Figure S1. Overview of compounds compared with Ni, Pd, and Pt complexes for NMR spectra characterization and IC50 comparison.[Hildebrandt, 2016a; Hildebrandt, 2022]

Stability Determination

Figures S2 and S3 show stability investigations for Ni2 and Pd3 by NMR and for Ni3, Pd3 and Pt3 by UV-VIS spectroscopy as examples for all discussed metal complexes.

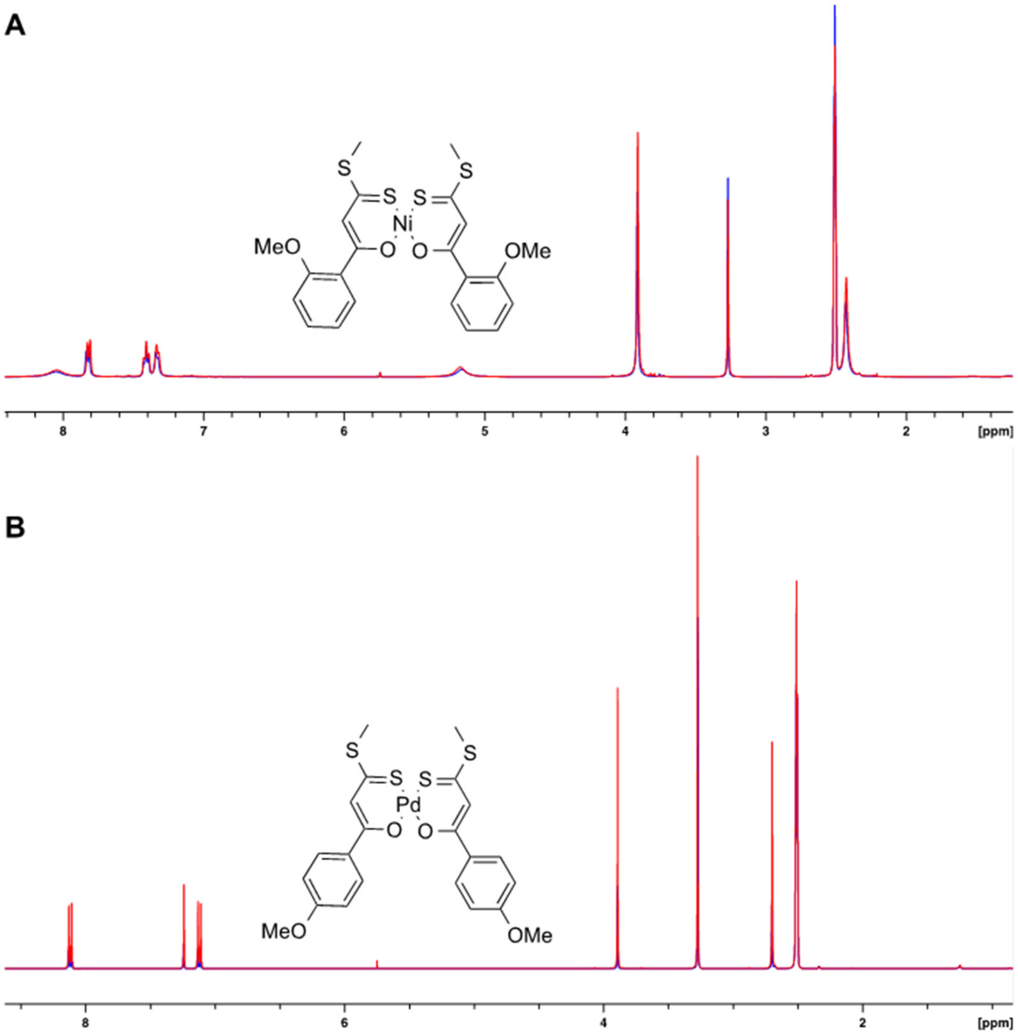

Figure S2. Stability determinations by ^{1}H NMR (400MHz) for Ni1 and Pd3 as examples for all complexes discussed in this work. Conditions: dmso-d_6 as solvent, 48 h measurements (red: t=0, blue: t= 48 h) at 37 °C.

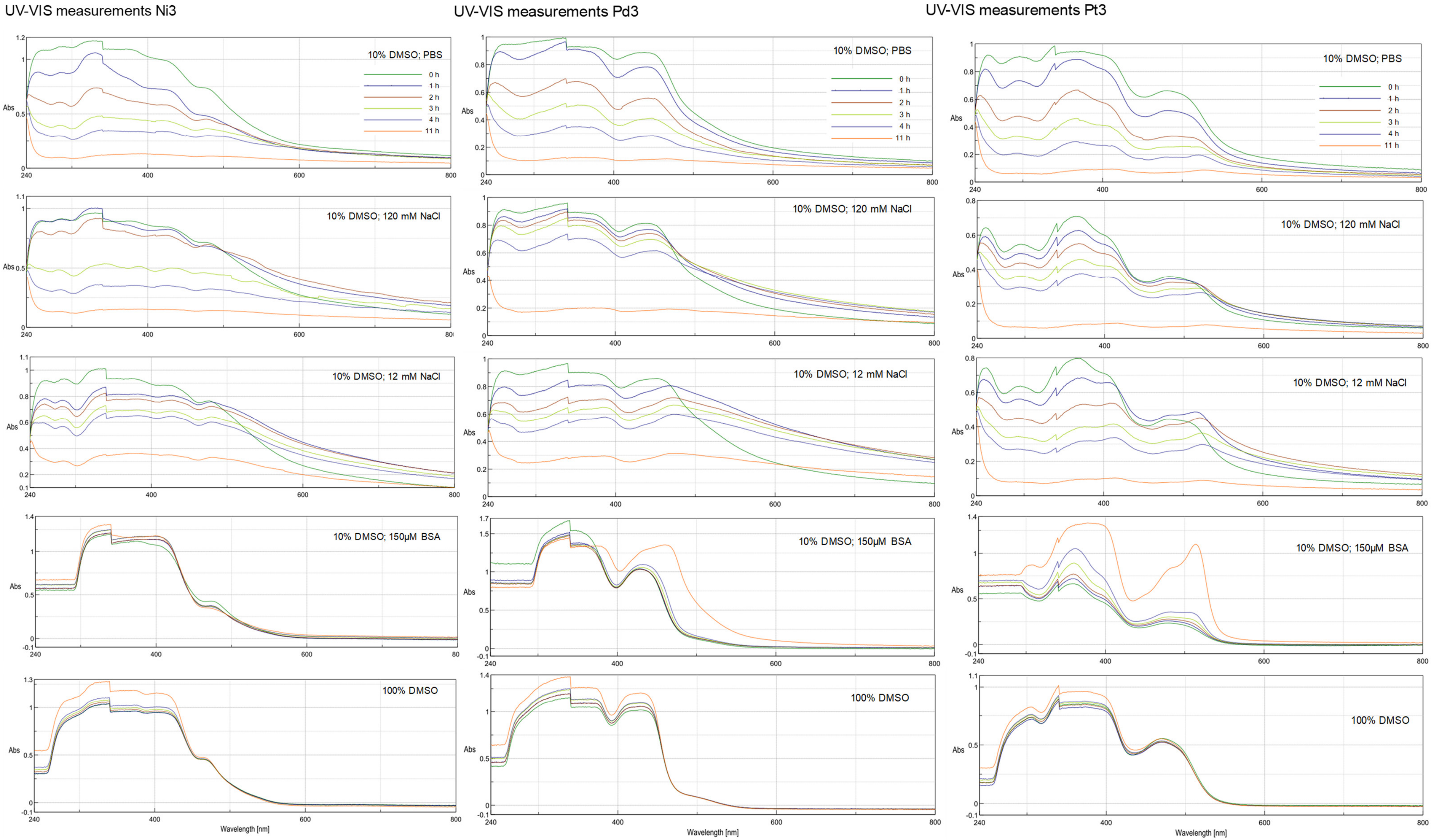

Figure S3. UV-VIS spectroscopy for Ni3, Pd3, Pt3 (100 µM) in different buffers over 11 hours. The change of the measured absorbance at 340 nm is caused by the change of the light source. The compounds are stable and soluble in DMSO they precipitate in 10 % DMSO with PBS and NaCl (12 mM, 120 mM). In 10 % DMSO with 150 µM BSA the compounds do not precipitate. Precipitation results in an overall decrease of absorbance.

Additional Molecular Structures

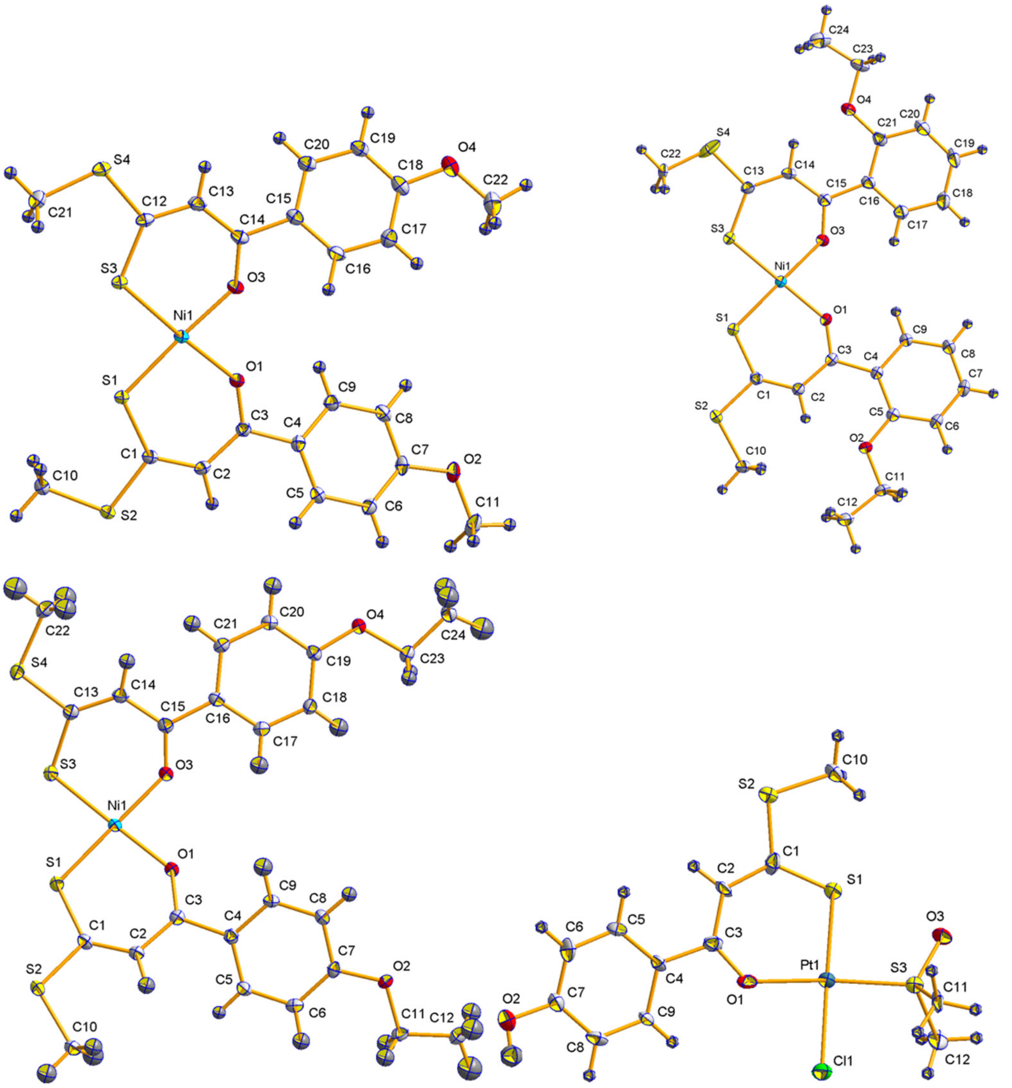

Figure S4. Molecular structures (50% probability) of Ni3, Ni4, Ni6 and Ptdmso8.

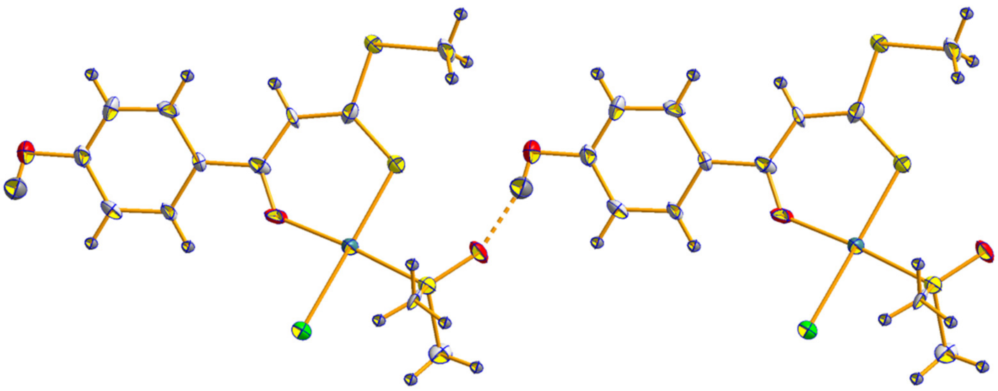

Figure S5. Intermolecular hydrogen-bonding observed in the crystals of Ptdmso8.

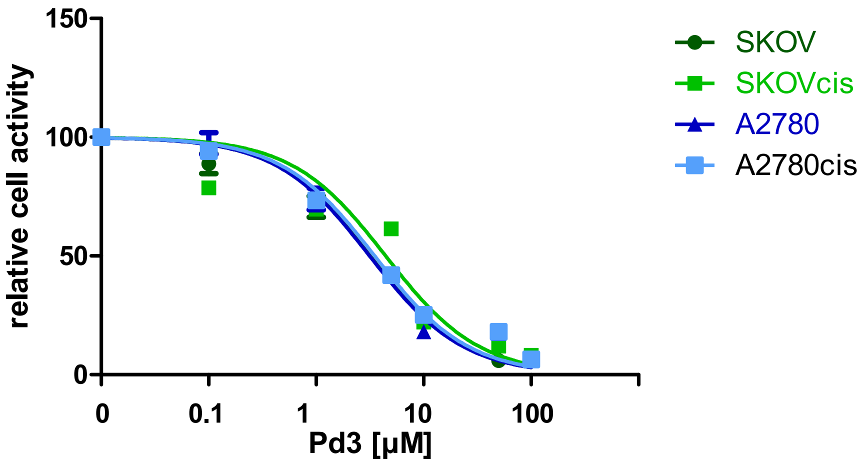

Figure S6. Exemplary dose-response curves for Pd3.

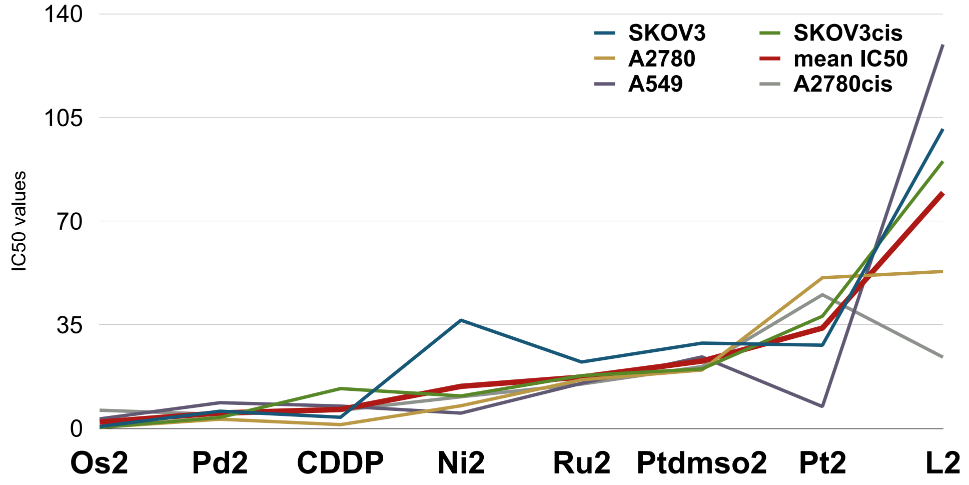

Figure S7. Influence of the metal for compounds number 2 on IC_{50} values. Substances were ordered with increasing mean IC_{50} values.

Table S1. Specific bond angles [°] and bond lengths [Å] for all characterized nickel(II) and palladium(II) compounds.

	**Ni1**	**Ni3**	**Ni4**	**Ni6**	**Pd1**
O(1)-C(3)	1.258(3)	1.270(2)	1.277(3)	1.269(3)	1.271(7)
C(3)-C(2)	1.398(3)	1.416(2)	1.415(3)	1.417(3)	1.400(9)
C(2)-C(1)	1.378(3)	1.374(2)	1.374(3)	1.366(3)	1.369(9)
C(1)-S(1)	1.698(2)	1.7075(18)	1.708(3)	1.711(2)	1.703(6)
O(1)-M(1)	1.8466(17)	1.8492(12)	1.8695(17)	1.8720(16)	2.023(4)
S(1)-M(1)	2.1429(7)	2.1434(4)	2.1309(7)	2.1426(6)	2.2307(16)
O(3)-C(14/15)	1.264(3)	1.278(2)	1.279(3)	1.268(3)	1.254(7)
C(14/15)-C(13/14)	1.386(3)	1.401(3)	1.411(4)	1.420(3)	1.510(9)
C(13/14)-C(12/13)	1.383(3)	1.386(3)	1.379(4)	1.367(3)	1.415(9)
C(12/13)-S(3)	1.696(2)	1.7085(18)	1.704(3)	1.720(2)	1.707(6)
O(3)-M(1)	1.8584(17)	1.8512(12)	1.8668(18)	1.8827(15)	2.049(4)
S(3)-M(1)	2.1426(7)	2.1406(5)	2.1396(7)	2.1510(6)	2.2348(16)
O(1)-C(3)-C(2)	123.5(2)	124.45(16)	123.5(2)	125.2(2)	127.0(6)
C(3)-C(2)-C(1)	125.7(2)	124.65(16)	124.5(2)	125.3(2)	128.0(6)
C(2)-C(1)-S(1)	128.83(19)	128.24(13)	129.1(2)	128.21(17)	130.4(5)
S(3)-C(12/13)-C(13/14)	128.19(19)	128.26(14)	128.9(2)	127.31(18)	131.5(5)
C(12/13)-C(13/14)-C(14/15)	125.3(2)	124.91(17)	125.2(2)	125.4(2)	121.1(5)
C(13/14)-C(14/15)-O(3)	126.0(2)	124.35(17)	123.8(2)	124.9(2)	124.8(6)
M(1)-O(1)-C(3)	136.99(17)	136.96(11)	137.39(16)	134.51(14)	129.9(4)
O(1)-M(1)-S(1)	96.42(6)	95.47(4)	95.22(6)	96.44(5)	96.78(13)
S(3)-M(1)-O(3)	97.07(5)	95.76(4)	95.96(6)	95.37(5)	94.93(13)
C(14/15)-O(3)-M(1)	133.89(17)	36.84(12)	136.51(17)	133.55(15)	132.9(4)
M(1)-S(3)-C(12/13)	108.84(9)	109.84(7)	109.60(9)	108.49(8)	107.5(2)
O(1)-M(1)-O(3)	80.14(7)	81.43(5)	84.06(8)	83.38(7)	83.26(17)
S(1)-M(1)-S(3)	86.46(3)	87.334(17)	84.86(3)	84.84(2)	85.08(6)
O(1)-M(1)-S(3)	176.48(6)	176.99(5)	177.73(6)	177.53(5)	176.20(13)
S(1)-M(1)-O(3)	175.71(6)	176.90(4)	177.55(7)	179.16(6)	179.22(13

Table S2. Specific bond angles [°] and bond lengths [Å] for Ptdmso8.

O(1)-Pt(1)	2.014(10)
S(1)-Pt(1)	2.258(3)
Cl(1)-Pt(1)	2.337(3)
S(3)-Pt(1)	2.215(3)
O(1)-C(3)	1.291(15)
S(1)-C(1)	1.692(13)
O(2)-C(7)	1.365(17)
O(1)-Pt(1)-S(3)	174.9(3)
S(3)-Pt(1)-Cl(1)	90.56(12)
Cl(1)-Pt(1)-O(1)	84.9(3)
S(1)-Pt(1)-S(3)	88.75(12)

Table S1 displays an overview of characteristic bond lengths and angles for bischelating compounds, Figure S3.

Table S2, Figure S3 and Figure S4 shows compound Ptdmso8. All data is in good agreement with those reported earlier for similar complexes and those which has been discussed in the main part of the manuscript.[Hildebrandt, 2016a] Compound Ptdmso8 shows intermolecular hydrogen bonding, as it has been already shown for Ptdmso10.[Hildebrandt, 2016a]

Table S3. Crystal data and refinement details for the X-ray structure determinations of the compounds **Ni1 - Ni4**.

Compound	**Ni1**	**Ni3**	**Ni4**
formula	$C_{22}H_{22}NiO_4S_4$	$C_{22}H_{22}NiO_4S_4$	$C_{24}H_{26}NiO_4S_4$
fw ($g\cdot mol^{-1}$)	537.35	537.35	565.40
°*C*	-140(2)	-140(2)	-140(2)
crystal system	triclinic	monoclinic	triclinic
space group	$P\bar{1}$	$P2_1$	$P\bar{1}$
a/ Å	8.9141(6)	12.0833(2)	8.1607(2)
b/ Å	11.1045(8)	7.0305(2)	10.4617(4)
c/ Å	11.9011(9)	13.5338(4)	15.0239(5)
α/°	84.903(4)	90	78.659(1)
β/°	84.716(3)	97.701(1)	88.945(2)
γ/°	71.651(2)	90	74.336(2)
$V/Å^3$	1111.17(14)	1139.35(5)	1210.09(7)
Z	2	2	2
ρ ($g\cdot cm^{-3}$)	1.606	1.566	1.552
μ (cm^{-1})	12.77	12.45	11.77
measured data	14108	8607	15199
data with $I > 2\sigma(I)$	3432	4989	4661
unique data (R_{int})	3691/0.0496	5079/0.0227	5524/0.0341
wR_2 (all data, on F^2)[a)]	0.1030	0.0569	0.0897
R_1 ($I > 2\sigma(I)$) [a)]	0.0402	0.0235	0.0432
S [b)]	1.067	1.064	1.075
Res. dens./$e\cdot Å^{-3}$	0.741/-0.749	0.378/-0.201	0.737/-0.785
Flack-parameter	-	0.152(10)	-
absorpt method	multi-scan	multi-scan	multi-scan
absorpt corr $T_{min/max}$	0.5782/0.7456	0.6651/0.7456	0.6506/0.7456
CCDC No.	1953242	1953243	1953244

Table S4. Crystal data and refinement details for the X-ray structure determinations of the compounds **Ni6 - Ptdmso8**.

Compound	**Ni6**	**Pd1**	**Ptdmso8**
formula	$C_{24}H_{26}NiO_4S_4$	$C_{22}H_{22}O_4PdS_4$	$C_{12}H_{15}ClO_3PtS_3$
fw ($g\cdot mol^{-1}$)	565.40	585.04	533.96
°*C*	-140(2)	-140(2)	-140(2)
crystal system	monoclinic	triclinic	monoclinic
space group	$P2_1/n$	$P\bar{1}$	$P2_1/c$
a/ Å	10.0852(2)	7.1500(3)	5.7783(6)
b/ Å	22.9804(6)	10.8504(5)	10.9133(12)
c/ Å	10.6285(3)	15.5213(7)	25.682(3)
α/°	90	78.967(2)	90
β/°	92.662(1)	85.536(3)	92.801(2)
γ/°	90	76.541(2)	90
$V/Å^3$	2460.62(11)	1148.73(9)	1617.6(3)
Z	4	2	4
ρ ($g\cdot cm^{-3}$)	1.526	1.691	2.193

μ (cm^{-1})	11.57	11.99	92.28
measured data	17207	8764	12971
data with I > 2σ(I)	4850	3852	3295
unique data (R_{int})	5545/0.0465	5013/0.0466	3606/0.0508
wR_2 (all data, on F^2)[a]	0.0775	0.1387	0.2394
R_1 ($I > 2\sigma(I)$) [a]	0.0366	0.0612	0.0745
S [b]	1.061	1.152	1.090
Res. dens./e·Å^{-3}	0.400/-0.321	0.751/-0.884	4.599/-3.298
absorpt method	multi-scan	multi-scan	multi-scan
absorpt corr $T_{min/max}$	0.6697/0.7456	0.6207/0.7456	0.5337/0.7456
CCDC No.	1953245	1953246	1953247

[a] Definition of the R indices: $R_1 = (\Sigma || F_o| - |F_c ||)/\Sigma | F_o |$;
$wR_2 = \{\Sigma[w(F_o^2-F_c^2)^2]/\Sigma[w(F_o^2)^2]\}^{1/2}$ with $w^{-1} = \sigma^2(F_o^2) + (aP)^2+bP$; $P = [2F_c^2 + Max(F_o^2]/3$;
[b] $s = \{\Sigma[w(F_o^2-F_c^2)^2]/(N_o-N_p)\}^{1/2}$.

Referenzen

[Hildebrandt 2016a] J. Hildebrandt, N. Häfner, H. Görls, D. Kritsch, G. Ferraro, M. Dürst, I. B. Runnebaum, A. Merlino and W. Weigand, Dalton transactions, 2016, 45, 18876-18891.

[Hildebrandt 2022] J. Hildebrandt, N. Häfner, D. Kritsch, H. Görls, M. Dürst, I. B. Runnebaum and W. Weigand, submitted, 2022.
